# Feasibility, acceptability, and adherence of two educational programs for care staff concerning nursing home patients’ fecal incontinence: a pilot study preceding a cluster-randomized controlled trial

**DOI:** 10.1186/s13012-015-0263-8

**Published:** 2015-05-23

**Authors:** Lene Elisabeth Blekken, Sigrid Nakrem, Kari Hanne Gjeilo, Christine Norton, Siv Mørkved, Anne Guttormsen Vinsnes

**Affiliations:** Faculty of Nursing, Sør-Trøndelag University College, Trondheim, Norway; Department of Public Health and General Practice, Norwegian University of Science and Technology, Trondheim, Norway; Department of Cardiothoracic Surgery, Department of Cardiology and National Competence Centre for Complex Symptom Disorders, St. Olavs Hospital, Trondheim University Hospital, Trondheim, Norway; Department of Circulation and Medical Imaging, Faculty of Medicine, Norwegian University of Science and Technology, Trondheim, Norway; Faculty of Nursing and Midwifery, King’s College London, 57 Waterloo Road, London, SE1 8WA UK; Clinical Service, St. Olavs Hospital, Trondheim University Hospital, Trondheim, Norway

**Keywords:** Fecal incontinence, Nursing homes, Long-term care, Older patients, Implementation study, Pilot study, Feasibility study, Care processes, Nursing

## Abstract

**Background:**

Fecal incontinence has a high prevalence in the nursing home population which cannot be explained by co-morbidity or anatomic and physiological changes of aging alone. Our hypothesis is that fecal incontinence can be prevented, cured, or ameliorated by offering care staff knowledge of best practice. However, it is not clear which educational model is most effective. To assess the effect of two educational programs for care staff, we planned a three armed cluster-randomized controlled trial. There is a lack of research reporting effects of interventions targeting improved continence care processes in older patients. Thus, to improve the quality of the planned trial, we decided to carry out a pilot study to investigate the feasibility of the planned design, the interventions (educational programs) and the outcome measures, and to enable a power calculation. This paper reports the results from the pilot study.

**Methods:**

Three nursing homes, representing each arm of the planned trial, were recruited. Criteria for assessing success of feasibility were pre-specified. Methods, outcome measures, acceptability, and adherence of the components of the intervention were evaluated by descriptive statistical analyses and qualitative content analysis of one focus group interview (*n* = 7) and four individual interviews.

**Results:**

The main study is feasible with one major and some minor modifications. Due to challenges with recruitment and indications supporting the assumption that a single intervention with one workshop is not sufficient as an implementation strategy, the main study will be reduced to two arms: a multifaceted education intervention and control. The components of the multifaceted intervention seemed to work well together and need only minor modification. Important barriers to consider were sub-optimal use of skill-mix, problems of communicating important assessments and care plans, and isolated nurses with an indistinct nurse identity.

**Conclusions:**

Overall, the main study is feasible. The pedagogical approach needs to consider the identified barriers. Thus, it is essential to empower nurses in their professional role, to facilitate clinical reasoning and critical thinking among care staff, and to facilitate processes to enable care staff to find, report, and utilize information in the electronic patient record.

**Trial registration:**

ClinicalTrials.gov: NCT01939821

## Background

Fecal incontinence (FI) is defined by the International Consultation on Incontinence as “the involuntary loss of liquid or solid stool that is a social or hygienic problem” [1]. FI has a higher prevalence in the nursing home (NH) population than in younger people, which cannot be explained by co-morbidity or anatomic and physiological changes of aging alone [2]. In the NH population, previous studies suggest prevalence between 10 and 69 % [3–5], most often reported to be between 40 and 55 % [5–8]. FI is associated with shame, social isolation, and reduced quality of life [1, 9, 10]. FI leads to a high direct and indirect economic burden to the health-care system and is an important cause of institutionalization of elderly patients [2, 7].

Among older patients, FI has a more complex etiology compared to the younger population [2]. Examples of reversible risk factors are loose stool, impaction, medication, inappropriate laxative use, toilet access, and quality of continence care [1, 8, 11]. Use of incontinence pads and toileting programs comprise the most common management in long-term care settings [12–14]. The level of awareness among health-care personnel regarding appropriate assessment and treatment options seems limited [1, 15, 16]. The hypothesis of this study is that FI among NH patients can be prevented, cured, or ameliorated by offering care staff knowledge of best practice.

There is a substantial evidence base to guide choice of implementation activities targeting health-care professionals in general [17–20]. However, relatively little of the implementation research has focused on care processes among older patients in NHs [21]. Specifically, there are few trials on either treatment of FI in NH patients nor on continence education programs for care staff [1]. Thus, we planned a three armed cluster-randomized controlled trial (C-RCT), with the aim to evaluate the effect of two educational programs with different degrees of complexity for care staff. Implementation research recommends multifaceted strategies to promote change of practice. In addition, it is important to investigate potential barriers [17, 19, 22]. Our rationale for choosing an interactive educational program was based on recommendations from the International Continence Society on the need to educate health-care providers to heighten awareness of FI, plus methods of identification, assessment, and management in older people [1]. The researchers’ competence in educational theory and delivery competence was also an important rationale. To improve the quality of the planned C-RCT, we decided to carry out a pilot study to investigate the feasibility of the planned design, the interventions (educational programs), and the outcome measures.

The specific aims of the pilot study were to evaluate feasibility, acceptability, and adherence to the educational interventions and methods used. The UK Medical Research Council (MRC) [23] defines an educational intervention as a complex intervention; hence, an essential purpose was to investigate whether all the components could work together. Even though the pilot is a small study, the results will be used to inform an estimate of the intra-cluster correlation coefficient (ICC) and inform estimation of sample size for the main study.

## Methods

The pilot study was designed as an external pilot which is a small-scale version of the main study which is not intended to be a part of the main study [24]. The pilot intervention period was 3 months. The study design was based on published guidance for developing and testing complex interventions [23–25].

### Setting

In Norway, most NHs are owned and run by the municipalities and financed by taxes and patient payment. A majority of the patients are above 67 years, have complex health problems, significant deficiencies in functioning related to activities of daily living (ADL), and about 80 % suffer from cognitive impairment [26]. There are no legal requirements for staff-to-patient ratios or specifications of qualifications required for workers [27]. However, NHs have RNs on duty 24 h a day. In addition, NH staff may comprise some authorized social educators (ASE) who have a bachelor’s degree in care related to people with intellectual disability, including dementia. ASEs have a defined health-care and pharmacological competence. According to Statistics Norway, the staff comprises on average 31 % RNs, 45 % licensed practical nurses (care education on a high school level most often leaving before the age 18), and 24 % health-care aides (no vocational health education). Statistics Norway has overall responsibility for official statistics in Norway.

### Participants

The sample was recruited from the same urban municipality in Norway as intended for the C-RCT. The municipality has a total of 27 NHs. All NHs are under the administration of the director for health and social affairs in the municipality. NHs are typically managed by registered nurses (RNs) and have an agreement with a general practitioner (GP) who visits the NH once a week. Under the manager, a NH may have one or several care managers. The care managers are most often not involved in the everyday care of patients. We recruited three NHs for the pilot, representing each arm in the planned C-RCT. These NHs have 24-h long-term residency, were recruited based on the same eligibility criteria as for the planned C-RCT and allocated as a cluster to single intervention (SI), a multifaceted intervention (MI), or control (C). NHs with similar staff-to-patient ratios on the day shift and GP coverage were eligible for selection. NHs designated with a specialty or with enhanced staff-to-patient ratio were excluded. RNs/ASEs working half time or more were eligible for participation in the workshop (see below) and to be recruited as an opinion leader (see below) in the intervention group. RNs/ASEs working less than half time or only night shifts were excluded. For the pilot, only RNs were involved in the study, and for the rest of the text we will use the term RN only. All care staff members in the NH were invited to the educational outreach meetings (see below) throughout the intervention period. All long-term care patients (who had stayed one month or more) were eligible for inclusion.

### Intervention

The educational programs were developed according to recommendations from implementation research, pedagogic theory, and experience from members in the project group [17–22, 28–32]. To ensure a realistic intervention, one of the researchers had two meetings with experienced NH nurses to collect their comments on content and intensity of the educational programs and on the FI guideline.

#### The FI guideline

The project group developed a FI guideline for nurse-led assessment and treatment of FI based on international best practice recommendations [1, 33–35]. The FI guideline facilitates a systematic assessment and includes questions related to bowel symptom history and bowel patterns. As FI among NH patients is considered to have a complex etiology, the guideline facilitates the RNs to consider a range of possible causes. Examples are loose stools, immobility, cognitive impairment, impaction, and use of laxatives. Based on this assessment, the RN defines a nursing diagnosis, for example: FI related to loose stools, possibly due to incorrect doses of Laxoberal® (sodium picosulfate), urgency, and reduced mobility. This leads to FI episodes with loose stool and red perineal skin. The guideline then offers a range of possible interventions. An important intention is to empower the RNs’ clinical reasoning [36] and critical thinking [37, 38]. Individualization of the nurses’ diagnoses and the interventions for each patient is important. Both NHs receiving the SI and MI were introduced to the FI guideline during the workshop.

The SI comprised: one educational meeting (7 h), defined by the Cochrane Effective Practice and Organization of Care (EPOC) as “participation of health-care providers in conference, lectures, workshops, or traineeships” [19]. The educational meeting was organized as an interactive workshop that targeted knowledge, attitudes, and skills. The workshop was conducted in a meeting room in the NH. The workshop started with the RNs completing a knowledge test and was a part of the data collection and one of the outcome measures. However, by organizing it as a part of the workshop, the pedagogical intention was to make it a trigger for learning as answers were given in the following educational session. Part two of the workshop was case-based discussions concerning the FI guideline. How to integrate the use of the guideline to the electronic documentation system was an important issue. This was addressed by having access to a “learning module” in their local electronic patient record (EPR). Real patient cases were discussed, and the result was input into the EPR during the workshop. This gave the RNs and the care leaders the opportunity to experience how it could best be done. The topics of the workshop, including the guideline, were made available for the RNs as printed educational material.

In addition, MI comprised of two more elements: 1) recruitment of a local opinion leader, defined by EPOC as “use of providers nominated by their colleges as educationally influential” [19], and 2) educational outreach visits defined by EPOC as “use of a trained person who meets with providers in their practice setting to give information with the intent of changing the providers’ practice” [19]. The local opinion leader was recruited after the educational meeting based on the informant method [39]. This was done by discussing with the care manager which of the RNs was considered to be able to influence and motivate the staff in general. The care manager had the responsibility for facilitating adherence to the program and the guidelines in cooperation with the opinion leader. The local opinion leader and care manager received a 1.5-h additional educational meeting on how to fulfill their roles in the study. The opinion leader and the care manager received contact information for the researcher for support during the intervention period.

The educational outreach visits were carried out in the NHs, facilitated by the project coordinator, and consisted of six sessions, lasting 1.5 h each. The opinion leader prepared cases for discussion together with the project coordinator. The project coordinator is the first author of this article and is a RN with additional educational theory and delivery competence. All of the care staff were the target group for the educational outreach and were invited to participate in the educational meetings throughout the intervention period. Facilitating and empowering care staffs’ clinical reasoning and critical thinking were the main pedagogical approach.

#### Control group

The control group did not receive any educational program and continued with ordinary practice. The main reason for including a control group in the pilot study was to investigate their motivation to fill in questionnaires without getting the educational intervention in return.

### Measures

The overall aim for the C-RCT is to study the effect of offering NH care staff an educational program on diagnosing and treating FI on reduction in FI for NH patients. The C-RCT primary outcome is frequency of FI among patients, and secondary outcomes are: remission of FI among patients identified with FI at baseline; incidence of FI among patients identified as continent at baseline; change in related concerns among patients; change in knowledge among RNs; and change in behavior among care staff. We also want to investigate correlates of FI among patients.

The following measures and data collection procedures were piloted for the main study:

The main unit of analysis for the planned C-RCT will be nursing home patients, and the same unit was used in the pilot study. The primary outcome measure was frequency of FI, measured by the Norwegian interRAI Long-Term Care Facilities Assessment System (interRAI LTCF) [40], section H3: Bowel continence. Bowel continence has the categories 0–5 where 0 = continent, 1 = continent with a stoma, 2 = seldom incontinent (not incontinent during the last three days, but has episodes of incontinence), 3 = occasionally incontinent (more seldom than daily), 4 = often incontinent (daily, but has some control), 5 = incontinent (no control), and 8 = did not occur (no bowel movement). The interRAI is a standardized, validated and comprehensive tool to assess patients’ health status. It measures patients’ functional, medical, cognitive, and psychosocial status [40]. In order to get some additional information on type of FI (gas, loose, or solid stool), urgency, and impact on daily life, a Norwegian version of the St. Marks anal incontinence score [41] was used. It gives a total score from 0 (complete continence) to 24 (complete incontinence).

Secondary outcome measures:

1. Both remission and incidence of FI measured by interRAI LTCF, section H3: Bowel continence.
2. Change in related factors measured by interRAI LTCF, section E: Mood and behavior, section F: Psychosocial well-being, section H1: Urinary continence, section J: State of health—Constipation and diarrhea, section L: Skin condition, and section M: Participation in activities.
3. Change in knowledge among RNs measured by a multiple choice test developed by the researchers according to established guidelines [42].
4. Change in care as reported in the EPR by care staff as measured by N-Catch. N-Catch is a validated audit instrument for care staff reports in the EPR [43–46]. N-Catch measures the quality of the content in the EPR on a scale from 0 to 32 where 0 is low quality and 32 is high quality. The instrument includes criteria for both quantity and quality of content. In order to get a score on quantity, the different parts only need to be present in the EPR (health status and a nursing care plan including nursing diagnoses, outcome, interventions, and evaluations). To get a high quality score, the content is assessed according to criteria reflecting clinical reasoning and critical thinking: does the assessment of health status seem sufficient, do the nurses’ diagnoses have a logical focus and etiology, and are the outcomes and interventions individualized, relevant, and realistic [34–38, 43–46]. Change in care will also be measured by the Fecal Incontinence in Nursing Home questionnaire [8] where RNs are offered a list of interventions relevant for FI and asked to identify what is done for each individual patient.

In addition, correlates of FI were measured by interRAI LTCF, section C: Cognitive functioning, section D: Communication and vision, section G: Functionality and mobility, section I: Medical diagnosis, section J: Health condition, section K: Mouth and nutrition status, section N: Medications, and section O: Treatment, examinations/procedures.

In the pilot study, the project coordinator gave information and training on completion of the interRAI [40], the St. Mark’s anal incontinence questionnaire [41], and the Fecal Incontinence in Nursing Home Patients questionnaire [8]. In addition, the project coordinator gave information and training on the procedure of printing data from the EPR in accordance with the audit instrument N-Catch. RNs with good knowledge of the patients completed questionnaires regarding patients’ health.

### Criteria for feasibility, adherence and acceptability

Feasibility, adherence, and acceptability of the educational programs were evaluated according to the following criteria:

Feasibility criteria:

1. Acceptable recruitment process.
2. >80% completed questionnaires returned
3. <10% missing data in each completed questionnaire
4. >0.5 mean change on the frequency scale on the primary outcome measure
5. Acceptable time use for RN’s involved in the data collection

Adherence criteria:

6. >95 % of the recruited RNs participated in the workshop
7. >70 % of the health personnel participated in the educational outreach on each actual day
8. >90 of the patients assessed by the FI-guideline
9. >80% of the assessment specified by the FI-guideline reported in the EPR

Acceptability criteria:

10. Acceptable performance of the knowledge test according to sensitivity to change in knowledge
11. Satisfaction from RNs regarding the educational intervention
12. Satisfaction and acceptability from RNs regarding the FI guideline
13. Acceptable level of barriers versus facilitators for change in the NHs

Quantitative data was collected at baseline (t_0_) and after 3 months (t_1_ = end of intervention). In order to obtain data concerning criteria 11–13, qualitative data was collected by one focus group interview [47, 48] performed 1 month after the end of the intervention. To receive additional information, four focused individual interviews [49] were performed 4 months after the end of the intervention. Informants were recruited from the two intervention NHs. The focus group interview was moderated by one of the researchers not involved in the intervention. The project coordinator was present and could ask questions to explore a theme. The individual interviews were performed by the project coordinator. All NHs were offered economic compensation linked to the data collection in order to pay for the process of hiring extra staff to make it possible for the RNs to be absent from daily care to undertake data collection.

### Analyses

Descriptive statistical analyses were performed using IBM SPSS version 21. Data from the interviews was digitally recorded, transcribed, and then analyzed by qualitative content analysis in accordance with Graneheim and Lundman [50].

All interviews were audiotaped and transcribed verbatim. First, the researcher reviewed the text several times to receive a general impression of the content. Second, the parts of the text addressing criteria 11, 12, and 13 were defined as content areas. Third, words, sentences, or paragraphs related to the content areas were identified and defined as meaning units. The meaning units were then condensed and labeled with a code. Fourth, the codes with similar meanings were grouped into categories. Related categories were then abstracted to themes with the intention to reveal the underlying meaning on an interpretive level [50]. The process from meaning units to themes went back and forth as members of the project group gave their feedback in the process of analysis.

### Ethical aspects

The study was conducted with the approval of the Regional Committee for Medical and Health Research Ethics (REK) (2013/755 REK Nord) and by The Norwegian Social Science Data Services (35020). NH managers were informed and gave permission to perform the study in the individual NH. Informed consent was obtained from RNs for the knowledge test. After evaluating the overall project, the REK authorized RNs recruited to be involved in the data collection procedure with dispensations from the duty of confidentiality to gather relevant patient health information (proxy data) in order to measure effect of the educational intervention. The patients were given written information about the study and had the opportunity to withdraw themselves from data being gathered. In cases where RNs assessed a patient as not cognitively competent to read and understand the information, the letter was sent to the patients’ representative. All patient information was de-identified by care staff before transfer to the researcher. The study was performed in concordance with the Helsinki Declaration. The project is registered in the clinical trial registry (NCT01939821).

## Results

The aims of the pilot study were to evaluate feasibility, acceptability, and adherence to the educational intervention and methods used.

### Recruitment

**Fig. 1:**
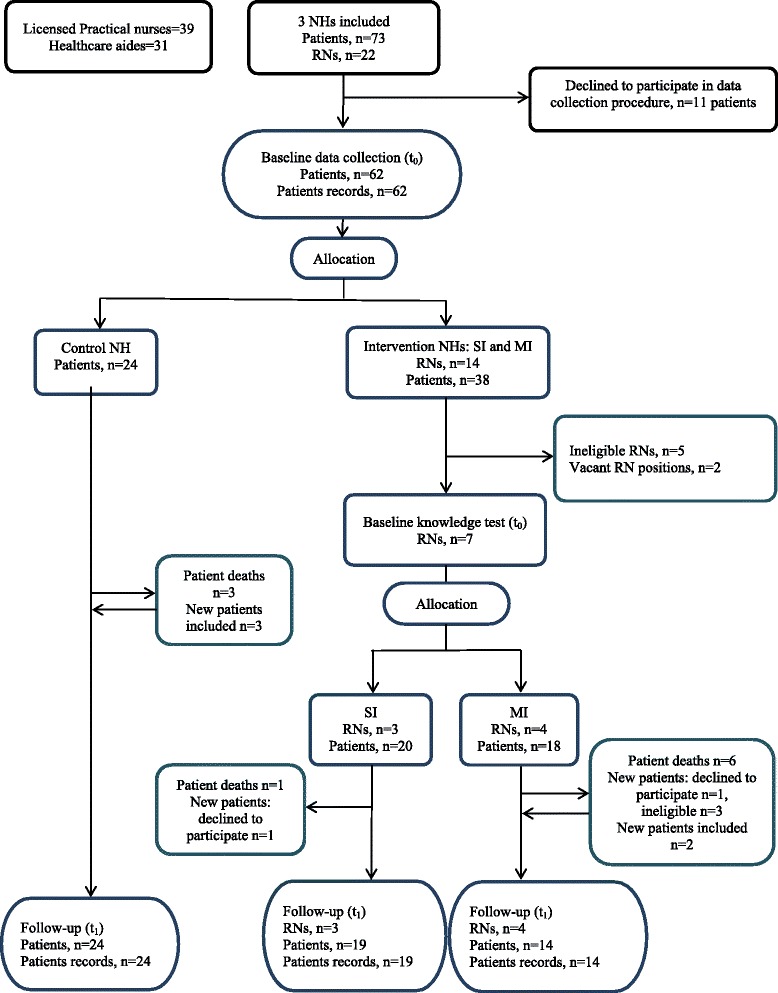
Flow chart of the inclusion and allocation of nursing homes

After obtaining approval from the director for health and social affairs in the municipality, an invitation letter was sent by email to the managers of 27 NHs. None of the managers responded positively to the first invitation. The project coordinator then telephoned the NH managers and asked if they were interested in participating. Three NHs were recruited (Table 1). The main reasons for declining were lack of time, that the NH was already involved in other time-demanding projects, and/or that the NH recently had major staff turnover. The process of recruiting NHs was challenging (criterion 1). For recruited NHs, all RNs with positions ≥50 % in the NHs were automatically recruited to participate in the workshop. Seven RNs were recruited to participate in MI (4) or the SI (3) (Table 2). Sixty-two patients participated in the baseline data collection, and 57 patients participated in the follow-up data collection (Fig. 1).

**Table 1:** Description of the nursing homes

	SI	MI	Control
Patient beds, long-term care	24	24	25
FTE^a^, RNs/ASEs, *n* (%)	6 (33)	5.6 (36)	7.95 (43)
Number of RNs employed	6	8	8
Number of ASEs employed	0	0	1
FTE^a^, licensed practical nurses, *n* (%)	7.21 (40)	8.5 (54)	9 (48)
Number of licensed practical nurses employed	11	13	15
FTE^a^, health-care aides, *n* (%)	4.81 (26)	1.4 (9)	1.7 (9)
Number of health-care aides employed	16	5	10
Number of formalized meetings with general practitioner, per week	1	1	1

*SI* nursing home receiving single intervention, *MI* nursing home receiving multifaceted intervention, *RN* registered nurses, *ASE* authorized social educators

^a^Full-time equivalent

**Table 2:** Demographics of the included nurses^a^

*n* = 7	SI	MI
Age, mean (range)	42 (36–48)	38.25 (23–52)
Sex	All female	All female
Years since graduated, mean (range)	12 (10–15)	9.8 (1–25)
Employed in this nursing home Years, mean (range)	2.4 (0,2–4)	5 (1–9)

*SI* nursing home receiving single intervention, *MI* nursing home receiving multifaceted intervention

^a^One of the included nurses in each nursing home had the position of care manager

### Data collection procedure

Two information meetings were arranged: one 1-h meeting regarding general information and the procedure for information distribution to patients or their representatives, and one 3-h meeting regarding the data collection procedure. Time spent on filling in the questionnaire at baseline was initially approximately 2 h per patient but reduced to approximately 1 h 15 min when RNs became familiar with the questionnaire. At follow-up, it was 45–60 min per patient. The interRAI questionnaire was the most time consuming. All RNs and care leaders involved in the data collection procedure reported the information process regarding data collection to be satisfactory and data collection to be time consuming. The project coordinator did not experience any challenges in the process of training RNs to collect data. The care leaders reported that the economic compensation provided was used to cover extra hired staff, so that the RNs responsible for the collection could withdraw from daily patient work. Even so, the RNs and the research team evaluated the use of time involved in the data collection procedure as unacceptable (criterion 5).

Time between delivering the questionnaires and completion was 18–28 days at baseline and 22–26 days at follow-up. Time frames included giving or sending out information letters to patients or their representatives. This time frame was evaluated as acceptable by the research team. Although time consuming, the project coordinator experienced NHs to be motivated to undertake the data collection at both baseline and follow-up, including the control NH. Research staff time for recruitment and follow-up was evaluated as acceptable (criterion 1).

The RNs filled in questionnaires for all of the patients who met the eligibility criteria and did not decline to participate. The proportion of missing data in each completed questionnaire was less than 10 %. The result is in concordance with criteria 2 and 3. Table 3 shows the characteristics of the included patients. The characteristics were similar to NH patients in other studies [8].

**Table 3:** Selected baseline patients’ characteristics

*n* = 62	Baseline values
Age, mean years (SD)	86 (10.14)
Gender, female, *n* (%)	48 (77.4)
Sum Barthel ADL score^a^, *n* = 57^b^, mean (SD)	10.07 (5.5)
Cognitive impairment	
Yes, *n* (%)	37 (59.7)
Partly, *n* (%)	13 (21)

^a^Barthels scoring form for functioning in activities of daily living, scoring range 0–20 where 0 = independent

^b^Missing data from one or more of the individual ADL score

### Attendance at the workshop

After baseline data gathering, one NH was randomly allocated as a control. The other two NHs received the workshop as part of SI or MI. At both intervention NHs, the attendance was 100 % of the RNs, a total of seven RNs. The result is in concordance with criterion 6.

### The knowledge test

All the included RNs completed the knowledge test both at baseline and at follow-up. Results from the knowledge test are presented in Table 4. The knowledge test was defined as acceptable according to criterion 10. This conclusion was based on an evaluation after feedback from the included RNs. Overall, the RNs found the questions relevant and meaningful. The project coordinator investigated whether the RNs answered correctly or incorrectly on the same questions that could indicate that the question was too hard or easy or not relevant. Overall, incorrect/correct answers varied between RNs. However, there were more incorrect answers among anatomy/physiology questions, than among questions related to continence care.

**Table 4:** Results of the knowledge test^a^ for nurses

	Baseline, *n* = 7	Follow-up, *n* = 7	Difference, points (%)
SI			
Mean points (range), *n* = 3	14.7 (11.5–18.5)	16 (10–21)	1.3 (5.0)
MI			
Mean points (range), *n* = 4	17.1 (17–17.5)	21.6 (19–26)	4.5 (17.3)

*SI* nursing home receiving single intervention, *MI* nursing home receiving multifaceted intervention

^a^Scoring range 0–26 points. Twenty-six multiple choice questions: all question, except one, actuated 1 point per correct answer. One question actuated 0.5 or 1 point

### Local opinion leader and attendance at the educational outreach

One NH, consisting of 2 units with 12 patients per unit, was allocated to MI. Investigations revealed that units in NHs in the municipality were comparable with the functional definition made by Norton et al. [51]: a geographical area in a facility, serving a population of patients while they reside there, with dedicated management, which is characterized by: 1) a regular group of care personnel who deliver the direct care and who work most of their shifts on one unit, 2) a care manager who is in charge of the whole unit but whose supervision may stretch over several units, and 3) a RN who oversees the unit on a shift-by-shift basis but whose supervision may stretch over several units. We agreed with the care manager on recruiting one local opinion leader with responsibility for both units. The mean number of care staff on a day shift was 5.5 (per 24 patients). Of the seven planned meetings, five meetings were completed. Due to organizational issues, the period of the pilot was 2.5 months instead of the planned 3 months. The NH could not find time for more than five meetings during the 2.5 months. A mean of 29 % of the staff participated in the educational outreach. Participants were the local opinion leader (all meetings), the care manager (two meetings), and one other RN (one meeting). The result is not in concordance with criterion 7.

### Assessment with FI guideline and documentation in EPR

The NH receiving SI managed to assess 50 % of the patients with the FI guideline, and 26 % of the assessments were reported as a health status/individualized care plan in the EPR. NH receiving MI managed to assess 96 % of the patients, and 93 % of the assessments were reported in the EPR as a health status/individualized care plan. Only the NH receiving MI managed to assess and document in concordance with criteria 8 and 9. Researchers found N-Catch to be a useful audit instrument.

### Primary outcome measure

Table 5 shows prevalence of FI in the different NHs and mean change on the frequency scale among patients with FI. Both intervention NHs show a tendency to reduced frequency of FI among patients. However, the reduction was smaller than specified in criterion 4.

**Table 5:** Distribution of continence scores among patients with FI

	Baseline			Follow-up			
	Prevalence^a^, % (*n*)	Mean^a^	SD	Prevalence^a^, % (*n*)	Mean^a^	SD	Mean change
SI	60 (12)	3.83	0.84	(53) 10	3.80	1.32	−0.03
MI	50 (9)	3.00	1.23	(57) 8	2.63	0.92	−0.37
Control	58 (14)	3.77	1.37	(67) 16	4.38	1.15	+0.57

*SI* nursing home receiving single intervention, *MI* nursing home receiving multifaceted intervention

^a^interRAI, H3 bowel continence (scores 2–5) 2 = seldom (have episodes, but not the last 3 days), 3 = occasionally (more seldom than daily), 4 = often (daily, have a curtain control), 5 = incontinent (no control). Patients with the scores 0 = continent, and 1 = continent with a stoma, are excluded

### Satisfaction and acceptability regarding the educational intervention

All RNs (seven) in the intervention NHs participated in the focus group interview. Four RNs, two from each intervention NH, participated in focused individual interviews. The results are presented in Table 6. The RNs found the workshop inspiring. It gave them the opportunity for professional discussions and raised consciousness of bowel problems in general and FI in particular in the NH population. The professional discussions about best practice for the individual patient were considered motivating for their nursing practice. The FI guideline was reported as a tool that made them stop and think in a systematic and critical way. RNs representing the NH receiving the MI reported the educational outreach as essential for a change in practice. Even though the NH receiving the SI did report some change in care for patients with FI, they did not manage to keep up the focus over time. Examples of barriers to change reported by RNs were sub-optimal use of skill-mix and many different care staff members resulting in problems spreading the information about assessments and care decisions to all care staff. These barriers were reported as the main reason why the patient did not get the care as intended.

**Table 6:** Results from qualitative content analysis of interviews

Content area	Categories	Themes
Workshop	Professional discussions	Professional discussions as inspiration for best practice
	Motivating	
	Sharing	
Local opinion leader	Collaboration	Valued and empowering role, but significant allies are essential
	Mastering	
Educational outreach	Enabling	Change require guidance over time, feedback and a sense of ownership
	Maintained focus	
	Monitoring	
FI guideline	Organizes knowledge	The FI guideline facilitates clinical and critical thinking
	Decision support	
	Comprehensive	
	Concrete and goal oriented	
	Made nurses think	
Barriers	Staff discontinuity	Hard to communicate important information to all
	Insufficient time	
	Large care staff	
	Few RNs	Isolated nurses and vague nurse identity in a fragmented care community
	Unclear nursing role	
	Sub-optimal use of skill-mix	
	Reluctance to use computers	Insecurity in how to find, report and utilize assessments and care plans in EPR
	EPR is difficult to navigate	
	Too few computers	
Facilitators	Demonstrable results	Raised consciousness on bowel problems and concrete results motivates
	Heightened awareness	
	Distinct nurse identity	Strong nurse identity in a positive care community
	Sense of community	

An important intention with the EPR is to make it possible for care staff to communicate their assessments and care plans as a means to secure continuity in care. RNs reported frustration with finding time to do the tasks involved in patient assessments and development of care plans, and if developed, that “nobody” read and followed the directions. Possible explanations described by all informants were lack of time and uncertainty on how to communicate and report care in the EPR, inefficient software, too few computers in the units, and a reluctance to use computers. RNs also described the nursing role as unclear based on the tendency to distribute tasks equally between staff irrespective of their level of qualification. This includes non-nursing tasks such as preparing food, washing patients’ clothes, and cleaning beds. The results from the interviews indicate that the intervention facilitated a stronger nurse identity and raised consciousness on the importance of assessments and individual care. According to the RNs, the input of knowledge and the use of the FI guideline led to demonstrable results; the patients experienced fewer episodes of FI, which worked as an important motivation for adherence to the care plan. RNs also reported the FI guideline as a tool which helped them structure bowel assessments, identify FI-etiology and intervention, and was used as a decision support in the process of documenting a care plan in the EPR. All informants considered a positive care community as essential for change. The results are in concordance with criteria 11 and 12. Results related to criterion 13 will be used to tailor the intervention to overcome the identified barriers.

## Discussion

The aims of the pilot study were to evaluate feasibility, acceptability, and adherence to the educational intervention and methods used. Overall, the pilot study showed a reasonable result, which will guide the main study. However, some modifications are needed.

As the main study plans to recruit from the same municipality, the recruitment problem experienced needs thorough consideration. The recruitment problem for the pilot might reflect a lack of motivation to participate in a pilot study where the presented aim was not considered clinical relevant. The RNs participating in the pilot recommended a recruitment strategy involving a clearer focus on FI and bowel problems as this is something considered clinically relevant. For the main study, we also plan to include personal meetings with the director of health and social affairs and the care managers of the NHs. We will also invite one of the RNs from the pilot study to share her experience and to answer questions about participating.

RNs did manage to fill in questionnaires for all included patients with less than 10 % missing data and to print and de-identify the information from the EPR within a reasonable time frame. This was also the case for the NH in the control group. The economic compensation and the recommendation of releasing the responsible RNs from daily work were reported as essential. Even so, the RNs recommended the research team to make the data gathering less time demanding. Completing interRAI was reported to be most time demanding, but as it is the instrument that has gone through the most thorough validation process and is in worldwide use, the project group considered it as essential, leading to the removal of the Fecal Incontinence in Nursing Home Patients questionnaire instead. In addition, RNs found the work of completing interRAI meaningful as the task included a time resource to sit down, discuss, and do a thorough assessment of the patients.

The work of printing data from the EPR was not considered time consuming, but the process of de-identifying the content was. After testing N-Catch on the pilot data, we considered daily evaluations over a period of 4 weeks as sufficient to audit the content. Therefore, the printing of daily evaluations will be reduced from the previous 12 to 4 weeks. The process of reporting complete health status, identifying accurate nurses’ diagnoses, outcomes, and interventions is considered to reflect RNs’ ability to use clinical reasoning and critical thinking [34–38]. Therefore, a systematic analysis of the nursing reports based on the N-Catch criteria can be used as a measure of clinical reasoning and critical thinking in the main study.

Overall, the components of the intervention seemed to work well together. The workshop was judged as feasible when all eligible RNs participated. The result is in concordance with other studies [17, 19], which reported workshops as feasible in most settings.

The local opinion leader worked with the rest of the staff on her own unit as recommended by Flodgren et al. [39]. However, both care manager and opinion leader reported that it was important to recruit one opinion leader per unit for the main study. The units have separate staff with different cultures, and it was challenging to fulfill the role as intended in two units. For the main study, the functional definitions of a unit made by Norton et al. [51] will be used as guide for recruitment of a local opinion leader and to inform the definition of cluster in the trial where one unit will be defined as one cluster. In addition, using the unit as a cluster will improve study feasibility by increasing the number of potential clusters, which impacts power more than increasing individuals enrolled [24].

For the educational outreach meetings, the intention was to include as many of the total care staff on duty as possible. Another intention was for the local opinion leader to prepare cases for discussion for the outreach meeting. This did not work as intended. The problem of involving more of the staff may be due to the practical issues on how to organize care staff between work tasks in the unit and the educational outreach meetings as it is impossible for all of the staff to leave the unit at the same time. Another reason might be that the project had decided that only RNs were to assess the patient with the FI guideline, and that the rest of the care staff was too little involved. There are few RNs, and to implement a new routine, it is important that as many as possible of the staff have ownership of the routine to be implemented [22, 28, 31]. For the main study, the RNs will maintain responsibility for FI assessment in order to reinforce the empowerment of the RNs to take the leading role in patient care but in closer cooperation with the licensed practical nurse with primary care responsibility for the patient. To accomplish this, the researcher should motivate the care staff present at the educational outreach meeting to make a reasonable work plan until the next meeting, including who of the licensed practical nurses is to be included in the work. This will also ease the local opinion leader’s responsibility to prepare cases for discussion between meetings. This procedure was piloted, and the RNs involved reported this to work better than the original plan. The care leader and the opinion leader reported most of the licensed practical nurses and health-care aides to be positive and engaged, especially when they experienced a change among some of the patients. However, they experienced a challenge reaching all care staff, especially those working for few hours. An important strategy for implementing use of the FI guideline was to integrate the intervention with the existing EPR system [52]. The study identified insecurity in how to report and utilize assessment and care plans in the EPR as an essential barrier to change. Therefore, it is important to facilitate NH unit-specific strategies to ensure continuity in FI care for the individual patient.

Only the NH receiving MI managed to fulfill criteria 8 and 9. Results from the interviews support the assumption that RNs were motivated by the educational outreach meetings where they, together with the researcher, agreed upon how to continue the work. An issue for the researcher was to empower the RNs’ critical thinking and highlight that making assessments is an important care task. These results are supported by studies indicating that a workshop alone is not sufficient [17, 19, 22], and that educational outreach meetings might be essential to improve the care delivered [18].

The interviews identified a culture where the role of the RN was unclear and that RNs were doing many non-nursing tasks. Ausserhofer et al. [53] found the same tendency among RNs in hospitals all over Europe. They also found that nursing care activities most left undone were developing or updating nursing care plans, adequately documenting nursing care and adequate patient surveillance. Together with a discontinuity among staff, this may lead to a tendency of “private practice” where the individual care staff member does what they find best on their individual shift. For the main study, empowering RNs in the nursing role and helping them find ways to best organize the work on their own unit and give feedback to the rest of the care staff will be important.

Concerning the small change in the chosen primary outcome measure of FI episodes, the result needs to be interpreted with caution due to the small sample size and high drop-out rate of patients in the NH receiving MI. However, the main purpose was to get information for the planned C-RCT to inform the estimation of sample size and decide a model of analyses. Results from the pilot study showed that the primary outcome was skewed to the right with most of the patients defined as continent with a score of 0. As a consequence, we found it reasonable to dichotomize the variable in order to investigate the proportion of patients moving from one category to another. After discussing the results, the cut-off was set between the scores 2 and 3 on the interRAI scale with the categories *seldom incontinent* with the scores 0–2, and *often incontinent* with the scores 3–5 (see Table 7). We hypothesized that a reasonable and clinically important effect size in the intervention group compared to the control group would be 15 % between the two groups in proportions with FI (score of 3–5). A thorough discussion of the sample size calculations and model of analyses are published elsewhere [54].

**Table 7:** Distribution of scores after dichotomization of the primary outcome variable

interRAI^a^				
	Baseline		Follow-up	
	Seldom	Often	Seldom	Often
	Incontinent	Incontinent	Incontinent	Incontinent
SI, *n* (%)	8 (40)	12 (60)	10 (55.6)	8 (44.4)
MI, *n* (%)	13 (72.2)	5 (27.8)	11 (78.6)	3 (21.4)
Control, *n* (%)	14 (58.3)	10 (41.7)	10 (41.7)	14 (58.3)

*SI* nursing home receiving single intervention, *MI* nursing home receiving multifaceted intervention

^a^interRAI: 0 = continent, 1 = continent with stoma, 2 = seldom (have episodes, but not the last 3 days), 3 = occasionally (more seldom than daily), 4 = often (daily, has a curtain control), 5 = incontinent (no control); dichotomized scale: 0–2 = seldom incontinent, 3–5 = often incontinent

Results from the interviews stated that RNs and care staff in general did not manage to keep up the focus on FI assessment and management after a single intervention with a workshop. Together with the recruitment problem experienced and methodological considerations related to the complexity and cost of doing a study with three arms [24], the main study will be reduced to two arms: MI and a control group.

The generalizability and transferability of the results may be biased by the fact that recruiting NHs was challenging, and that NHs recruited may be higher functioning than the NHs rejecting participation for reasons outside the scope of the eligibility criteria, for example: personal characteristics of RNs and care leaders, care staff attitudes toward research and organizational change. As a consequence for the main study, the different components of the MI will be the same for all included NHs, while pedagogical strategies may vary in order to target needs in the individual NH, for example, to facilitate NH unit-specific strategies to ensure continuity in FI care and empower RNs in the nursing role.

In the main study, we will also include measures of adherence. These are: 1) proportion of RNs within eligibility criteria participating in the workshop, 2) how many and who of the care staff participated in the outreach meetings, 3) proportion of intended outreach meetings held, 4) proportion of patients assessed with the FI guideline, and 5) proportion of assessments reported in the EPR as health status and nursing care plan. In addition, researchers will record their reflections from the educational meetings.

## Conclusions

The aims of the pilot study were to evaluate feasibility, acceptability, and adherence to the educational intervention and methods used. The components of the intervention seemed to work well together. The results of the pilot study shows that the main study is feasible with one major change and some minor changes. An essential pedagogical approach is to facilitate clinical reasoning and critical thinking among care staff and to empower the RNs in their professional role. Another important pedagogical approach is to facilitate processes among care staff on how to find, report, and utilize assessments and care plans in the EPR. If the result of the main study shows an effect, an interesting question for later studies is whether it is possible to achieve significant effect with less effort.

## Abbreviations

FI: fecal incontinence
NH: nursing home
RN: registered nurse
ASE: authorized social educator
C-RCT: cluster-randomized controlled trial
EPR: electronic patient record
MRC: Medical Research Council
ICC: intra-cluster correlation coefficient
ADL: activities of daily living
MI: multifaceted intervention
SI: single intervention
C: control
EPOC: Cochrane Effective Practice and Organization of Care
interRAI LTCF: The interRAI Long-Term Care Facilities Assessment System
GP: general practitioner
